# ExAutoGP: Enhancing Genomic Prediction Stability and Interpretability with Automated Machine Learning and SHAP

**DOI:** 10.3390/ani15081172

**Published:** 2025-04-18

**Authors:** Yao Rao, Lilian Zhang, Lutao Gao, Shuran Wang, Linnan Yang

**Affiliations:** 1 College of Big Data, Yunnan Agricultural University, Kunming 650201, China; 2 Yunnan Engineering Technology Research Center of Agricultural Big Data, Kunming 650201, China; 3 Yunnan Engineering Research Center for Big Data Intelligent Information Processing of Green Agricultural Products, Kunming 650201, China

**Keywords:** genomic selection, genomic prediction, automated machine learning, SHapley additive exPlanations, animal breeding, ExAutoGP

## Abstract

Machine learning has attracted much attention in the field of genomic prediction due to its powerful predictive capabilities, yet the lack of an explanatory nature in modeling decisions remains a major challenge. In this study, we propose a novel machine learning method, ExAutoGP, which aims to improve the accuracy of genomic prediction and enhance the transparency of the model by combining automated machine learning (AutoML) with SHapley Additive exPlanations (SHAP). To evaluate ExAutoGP’s effectiveness, we designed a comparative experiment consisting of a simulated dataset and two real animal datasets. For each dataset, we applied ExAutoGP and five baseline models—Genomic Best Linear Unbiased Prediction (GBLUP), BayesB, Support Vector Regression (SVR), Kernel Ridge Regression (KRR), and Random Forest (RF). All models were trained and evaluated using five repeated five-fold cross-validation, and their performance was assessed based on both predictive accuracy and computational efficiency. The results show that ExAutoGP exhibits robust and excellent prediction performance on all datasets. In addition, the SHAP method not only effectively reveals the decision-making process of ExAutoGP and enhances its interpretability, but also identifies genetic markers closely related to the traits. This study demonstrates the strong potential of AutoML in genomic prediction, while the introduction of SHAP provides actionable biological insights. The synergy of high prediction accuracy and interpretability offers new perspectives for optimizing genomic selection strategies in livestock and poultry breeding.

## Simple Summary

In recent years, machine learning has garnered significant attention in genomic selection. However, the inherent “black-box” nature of machine learning models often hinders their interpretability, making it challenging to understand their decision-making processes. In this study, we propose ExAutoGP, a novel genome prediction method that leverages automated machine learning (AutoML) to enhance predictive accuracy while improving model interpretability by integrating SHapley Additive exPlanations (SHAP). We evaluate the predictive performance of ExAutoGP against genomic best linear unbiased prediction (GBLUP), BayesB, support vector regression (SVR), kernel ridge regression (KRR), and random forest (RF) across three datasets. The results demonstrate that ExAutoGP consistently delivers robust and superior predictive performance across all traits. Furthermore, the SHAP method was applied to determine the importance of the features and interpret the ExAutoGP model.

## 1. Introduction

Genomic selection is an advanced breeding strategy that significantly enhances the efficiency of genetic improvement by leveraging genome-wide single nucleotide polymorphisms (SNPs) to estimate individual genomic breeding values (GEBVs) [1]. The concept of genomic selection initially emerged in animal breeding and has gained widespread adoption over the past decade, driven by the rapid decline in the cost of high-throughput genotyping and sequencing technologies. This approach has been successfully applied in both animal and plant breeding, demonstrating its effectiveness and broad applicability [2,3]. Compared to conventional breeding methods that rely on phenotypic selection or marker-assisted selection, genomic selection overcomes key limitations by enabling the prediction of breeding values for individuals that have not undergone phenotypic evaluation. It operates under the assumption that each quantitative trait locus (QTL) is in linkage disequilibrium with at least one marker within the genome, thereby enhancing the accuracy of breeding value estimation. In addition to accelerating genetic progress and increasing genetic gain, genomic selection significantly reduces breeding costs [4]. For instance, its implementation in dairy cattle breeding in 2008 led to an approximately 92% reduction in breeding costs compared to traditional progeny testing, while also eliminating the need for progeny testing sessions [5]. In American Holstein cattle, the annual genetic gain for yield traits doubled, increasing from approximately 50% to 100% [6]. Today, genomic selection is widely employed in large-scale livestock breeding programs, including those for pigs, sheep, and poultry, with more than 400 million animals genotyped to date [7]. The adoption of genomic selection has revolutionized both animal and plant breeding, fundamentally transforming genetic improvement strategies and accelerating breeding efficiency [8,9].

Currently, parametric methods are the dominant tools for genomic selection in practical breeding, mainly including GBLUP [10], single-step GBLUP [11], least absolute shrinkage and selection operator [12], and Bayesian methods, such as BayesB, BayesLasso [13,14]. GBLUP estimates GEBVs by integrating genome-wide marker data with phenotypic information [15], whereas Bayesian methods predict GEBVs by estimating the effects of each marker through a Bayesian strategy [16]. Usually, the prediction accuracy of Bayesian methods is better than that of GBLUP, but it relies on the Markov Chain Monte Carlo algorithm for parameter estimation, which has a high computational complexity and is analytically time-consuming. However, these methods are all based on linear models, which mainly consider additive genetic effects and ignore potentially complex non-linear relationships between markers and phenotypes, such as epistasis, dominance effects, and genotype–environment interactions [17]. In addition, these methods usually assume that genotypic and phenotypic data obey specific distributions (e.g., normal distribution) [1] and assume that the effects of each gene are independent and fixed, which fails to adequately capture interactions between genes and between genes and the environment.

To overcome the limitations of traditional parametric models, machine learning (ML) has garnered significant attention in genomic selection due to its ability to model complex nonlinear relationships. As a key branch of artificial intelligence, ML estimates GEBVs by uncovering statistical patterns between large-scale genotype and phenotype data without requiring strict assumptions about data distribution or gene effects [18]. Unlike conventional methods, ML can integrate genome-wide marker information and effectively capture nonlinear interactions in high-dimensional genomic data, making it particularly advantageous when the number of variables far exceeds the sample size. Furthermore, as an advanced branch of machine learning, deep learning employs multilayer neural networks to model complex nonlinear relationships. It is capable of automatically extracting features and uncovering deep patterns from high-dimensional genomic data. Consequently, an increasing number of genomic selection studies have begun to incorporate deep learning approaches [19,20]. In recent years, ML has emerged as a powerful tool in genomic selection, offering new perspectives for genomic prediction. Despite its advantages, the widespread adoption of ML in genomic selection still faces several challenges. For researchers without expertise in ML, model selection and hyperparameter optimization often rely on manual tuning, which is both computationally intensive and laborious. Furthermore, the effective application of ML techniques necessitates a deep understanding of the theoretical foundations, algorithmic properties, and appropriate application scenarios of different methods, thereby increasing the technical barrier to entry. Consequently, optimizing model selection and parameter tuning to enhance the usability and practical applicability of ML in genomic selection remains a critical area of ongoing research.

To address the above issues, the use of AutoML has been proposed to optimize the ML process. AutoML is an automated end-to-end machine learning process that automates key steps in model development, including data preprocessing, feature engineering, model selection, and hyper-parameter tuning, thereby accelerating model deployment and significantly reducing reliance on human intervention and expertise [21]. Among the existing AutoML frameworks, the Genetic Programming-based Tree Process Optimization Tool (TPOT) is a widely used approach. TPOT is capable of automatically exploring and evolving the machine learning process to find the optimal machine learning pipeline [22]. It has been shown that AutoML performs well in several domains, for example, the AutoML tool developed by Simone et al. [23] demonstrated higher reliability in predicting articular osteoarthritis and achieved excellent performance in predicting 90-day mortality after gastric cancer surgery [24]. Furthermore, Zhang et al. [25] proposed a model called CascadeDumpNet that combines deep learning and AutoML for open dump detection, which effectively eliminated false detections and improved accuracy. Nevertheless, there are still unfilled gaps in the application of AutoML in animal genomic selection. Furthermore, the ‘black-box’ nature of ML models makes it difficult to interpret the results, which limits researchers’ trust and application of the models [26]. In the field of genomic selection, model interpretability is paramount. When generating predictions or recommendations, it is essential to provide clear, transparent, and logically coherent justifications. This ensures that breeders can trust the model’s outputs and make well-informed breeding decisions. Therefore, we hypothesize that integrating AutoML with interpretability methods can not only enhance the predictive performance of the model but also improve its transparency and practicality.

Based on the above background, this study proposes a novel machine learning approach, ExAutoGP, which integrates AutoML with SHAP to improve the accuracy and interpretability of genomic prediction. First, we present the framework design of ExAutoGP in detail, including the automated modeling pipeline optimized by AutoML and the implementation of SHAP for feature interpretation. Second, we conduct a comparative experiment using one simulated dataset and two real animal datasets to systematically evaluate the predictive performance and computational efficiency of ExAutoGP against baseline models (GBLUP, BayesB, SVR, KRR, and RF). A five-times repeated five-fold cross-validation scheme is employed to ensure the robustness and reliability of the results. In addition, SHAP-based visual analysis is used to elucidate the decision-making process of ExAutoGP and clarify the specific contribution of each feature to the model output. Finally, we comprehensively analyze the experimental results and discuss the advantages and potential applications of ExAutoGP in genomic prediction. Overall, our research results provide valuable insights into genomic prediction and offer a promising direction for optimizing breeding strategies.

## 2. Materials and Methods

### 2.1. Datasets

To evaluate the performance of ExAutoGP in comparison with other methods, three publicly available datasets were used. In brief, the details of these datasets are as follows.

#### 2.1.1. Simulation Dataset

The first dataset analyzed in this study was obtained from the 16th QTL-MAS workshop dataset (http://qtl-mas-2012.kassiopeagroup.com/en/dataset.php), which was simulated by Usai et al. (2012) [27]. This publicly available dataset comprises 3000 training individuals (from generations 1–3) and 1000 validation individuals (from generation 4). To simulate genetic effects, 50 QTLs were randomly selected, and their effect sizes were generated based on a gamma distribution with a shape parameter of 0.42 and a scale parameter of 5.4. Three genetically related quantitative traits (T1, T2, and T3) were generated from these 50 QTLs based on genotype data from 3000 trained individuals. The genotype data include five chromosomes, encompassing a total of 10,000 SNPs. This dataset has been widely used to assess the performance of genomic prediction methods [28,29].

#### 2.1.2. German Holstein Cattle

The dataset used genotype data from 5024 German Holstein bulls, all genotyped by Illumina Bovine SNP50 BeadChip (https://www.g3journal.org/content/5/4/615.supplemental). After quality control [28], SNPs with call rates below 95%, minimum allele frequencies (MAF) < 0.01, and deviations from Hardy–Weinberg equilibrium (HWE, *p* < 1 × $10^{-4}$) were excluded, and 42,551 SNPs were ultimately retained for subsequent analysis. Based on previous studies [30,31], three traits were selected that represented different genetic constructs: milk yield (MY, kg), milk fat percentage (MFP, %), and somatic cell score (SCS). The MFP trait had a high heritability and was mainly determined by a single main effector gene (the DGAT1 gene) along with multiple small effector genes and genomic regulatory elements (GREs). The MY trait has medium heritability and its variance is influenced by DGAT1 and multiple small and medium effector genes and GREs. The SCS trait, on the other hand, is a low-heritability trait, influenced by many small effector genes and GREs, and its phenotypic values have been transformed by a normal distribution to facilitate subsequent analyses.

#### 2.1.3. Chicken Dataset

This study utilized a dataset of 1063 Rhode Island Red chickens published by Liu et al. [32] (https://figshare.com/articles/Genome-wide_Association_Analysis_of_Age-Dependent_Egg_Weights_in_Chickens/5844420), which includes both genotypic and phenotypic data for egg weight (EW). The experimental material was obtained from the 11th-generation purebred Rhode Island Red chicken population of Beijing Huadu Yukou Poultry Breeding Co. (Beijing, China). To investigate the genetic basis of egg weight, 1078 hens with well-documented pedigrees were selected and genotyped using the Affymetrix Axiom® 600K Chicken Genotyping Microarray. Egg weight measurements were recorded at seven time points, spanning from the onset of egg laying to 80 weeks of age. In terms of phenotypic characterization, the first egg weight (FEW) was defined as the weight of the first egg laid by each hen. Subsequent egg weights were measured at 28, 36, 56, 66, 72, and 80 weeks of age. During the early laying period (≤56 weeks), egg weights were recorded over two consecutive days, whereas in the later laying period (>56 weeks), data were collected over three consecutive days. These time points were chosen based on breeding objectives, and the mean egg weight over 2 or 3 days was used as the phenotypic value for each sample. Individuals with missing genotypic or phenotypic data were excluded from the analysis. Since the dataset underwent quality control in the original study, no additional filtering was applied in this study. The three datasets contain 13 traits and the statistical information for each dataset is listed in Table 1.

### 2.2. Genomic Prediction Models

#### 2.2.1. GBLUP

GBLUP uses genomic relationships to estimate the genetic value of an individual [33]. The statistical model of GBLUP can be written as:

(1) $$y_{c}=1\mu +Zg+e,$$

where $y_{c}$ is a vector of corrected phenotypes of genotyped individuals, μ is the overall mean, 1 is an *n*-dimensional vector with all 1’s in a column, *Z* is the correlation matrix between $y_{c}$ and *g*, and *g* is a vector of genomic breeding values. The assumed normal distribution followed is $g∼N(0,G\sigma_{g}^{2})$, where *G* is the genomic relationship matrix (G matrix) and $\sigma_{g}^{2}$ is the additive genetic variance. The residual effect vector (*e*) is distributed as $e∼N(0,I\sigma_{e}^{2})$, where *I* is a unit matrix and $\sigma_{e}^{2}$ is the residual variance. GBLUP is implemented using the R package ‘BGLR’.

#### 2.2.2. BayesB

BayesB is a random search variable selection method [1]. BayesB assumes that most segments have no effect for a particular trait, an assumption that is consistent with the properties of quantitative traits, while a few segments have an effect and differ in the variance of the effect. The prior assumptions in BayesB for SNP effects and their corresponding variances are as follows:

(2) $$\alpha_{j}|\pi ,\sigma_{j}^{2}∼\left(iid\right)\left\{\begin{array}{cc} N(0,\sigma_{j}^{2}) & \;1-\pi \\ 0 & \pi \end{array},\right.$$

and

(3) $$\sigma_{j}^{2}|v_{\alpha },S_{\alpha }^{2}∼\left(iid\right)\chi^{-2}\left(v,S\right),$$

where $\alpha_{j}$ is the *j*-th labeled effect, $\sigma_{j}^{2}$ is the labeled effect variance, *v* is the a priori degrees of freedom, and *S* is the a priori scaling parameter. Meuwissen et al. [1] proposed that when the a priori distribution of the effect variance is a scaled inverse chi-squared distribution, its posterior distribution is likewise a scaled inverse chi-squared distribution. That is, $P\left(\sigma_{\alpha_{j}}^{2}\right)=\chi^{-2}\left(v,S\right)$, where $\sigma_{\alpha_{j}}^{2}$ is the *j*-th SNP effect variance, and here, the prior distribution of the SNP effect, P(α), is normal, i.e., it obeys the distribution $N(0,\sigma_{j}^{2})$, thus defining the prior distribution of $\sigma_{j}^{2}$, $P(\sigma_{\alpha_{j}}^{2})$. Within the Bayesian inference framework, the scaled inverse chi-squared distribution serves as the conjugate prior for the variance parameter of the normal distribution, facilitating posterior derivation and sampling, thereby improving computational efficiency [34]. Its heavy-tailed property also allows for better modeling of the heterogeneity in SNP effect variances, aligning with the sparsity assumption in genomic data where a small number of loci exhibit large effects while the majority have effects close to zero. BayesB is implemented using the R package ‘BGLR’.

#### 2.2.3. SVR

Support vector regression (SVR) is a technique for solving regression problems based on the principle of support vector machine [35]. SVR is an application of SVM to regression dealing with quantitative responses. It uses linear or nonlinear kernel functions to map the input space (labeled dataset) to a higher-dimensional feature space [36], and performs modeling and prediction on the feature space. The model formulation of SVR can be expressed as:

(4) $$f\left(x\right)=\beta_{0}+h{\left(x\right)}^{T}\beta ,$$

where f(x) is the vector of predicted values, $h{\left(x\right)}^{T}$ is the kernel function, β is the weight vector, and $\beta_{0}$ is the deviation. Typically, the formal SVR is given by minimizing the following limiting loss function:

(5) $$\min_{\beta_{0},\beta }\frac{1}{2}{∥\beta ∥}^{2}+C\sum_{i=1}^{n}V_{\epsilon }\left(f\left(x_{i}-y_{i}\right)\right),$$

where *C* is a regularization constant controlling the trade-off between prediction error and model complexity, *y* is the vector of observations, $V_{\epsilon }$ is the ϵ-insensitive loss, and ∥·∥ is the norm in Hilbert space. The ϵ-insensitive loss is defined as:

$$V_{\epsilon }\left(r\right)=\left\{\begin{array}{cc} 0 & \mathrm{if}\;|r|<\epsilon \\ |r|-\epsilon , & \mathrm{otherwise} \end{array}\right.,$$

After optimization, the final form of SVR can be written as:

(6) $$f\left(x\right)=\sum_{i=1}^{m}\left({\hat{a}}_{i}-\alpha_{i}\right)k\left(x,x_{i}\right),$$

where $k\left(x,x_{i}\right)=\phi {\left(x_{i}\right)}^{T}\phi \left(x_{j}\right)$ is the kernel function and ${\hat{a}}_{i}$ is the Lagrange multiplier. In this study, the SVR method is implemented using the SVR function in Python’s Scikit-learn library (Version 1.6.0) by searching for the best kernel function and hyperparameters through a grid search.

#### 2.2.4. KRR

Kernel Ridge Regression algorithm (KRR) is a nonlinear regression method that combines the advantages of kernel techniques and ridge regression and adds a kernel function to the ridge regression algorithm, which can be effective in discovering the nonlinear structure of data [37]. The difference between kernel ridge regression and ridge regression is that kernel ridge regression utilizes the kernel technique to define a higher-dimensional feature space and then constructs a ridge regression model in the feature space [38]. The KRR prediction function can be written as follows:

(7) $$f\left(x_{i}\right)=k^{\prime }{\left(K+\lambda I\right)}^{-1}y,$$

where $k^{\prime }$ is a vector, $k_{i}=\left(x,x_{i}\right),\;i=1,2,3,\ldots ,n$, *n* is the total number of samples in the training set, *K* is the Gram matrix, λ is the regularization constant, and *I* is the unit matrix. KRR is significantly computationally efficient, and it can deal with larger-scale datasets [39]. At the same time, it performs L2 regularization on the data to prevent overfitting, and due to its regularization property, it is also robust to data containing noise. We optimized the hyperparameters using a grid search approach and implemented the Kernel Ridge Regression (KRR) method via the KernelRidge function in Python’s Scikit-learn library (version 1.6.0).

#### 2.2.5. RF

Random Forest (RF) is a widely used ensemble learning algorithm for predicting GEBVs. RF was first proposed by Breiman [40], which is essentially an ensemble of decision trees [41]. The decision tree is used as the basis to integrate the prediction results of each base learner to obtain the final result. Therefore, the key step in the prediction of RF is the formation of decision trees and forests [42]. Random forests can handle a large number of features and are commonly used in classification and regression problems for high-dimensional data. In genomic selection, because the construction process of a random forest has randomness and uses random samples and random features, it has good robustness and can effectively avoid the problem of overfitting [43]. However, the higher the number of decision trees and the larger the depth of the tree in the random forest model, the higher the computational complexity, and a large amount of training data is needed to achieve better prediction results. The expression of random forest regression is:

(8) $$y=\frac{1}{M}\sum_{m=1}^{M}t_{m}\left(\psi_{m}\left(y:X\right)\right),$$

where *y* is the predicted value of the random forest regression, the predictor variable $t_{m}\left(\psi_{m}\left(y:X\right)\right)$ is the decision tree, constructed at the *m*-th iteration using bootstrap samples of the data $\psi_{m}\left(y:X\right)$, and *M* is the number of decision trees in the random forest. The RF method was implemented using the RF function from Python’s Scikit-learn library (Version 1.6.0), with a grid search employed to optimize the number of decision trees (M) and the maximum tree depth.

#### 2.2.6. TPOT: ExAutoGP Framework

**Figure 1:**
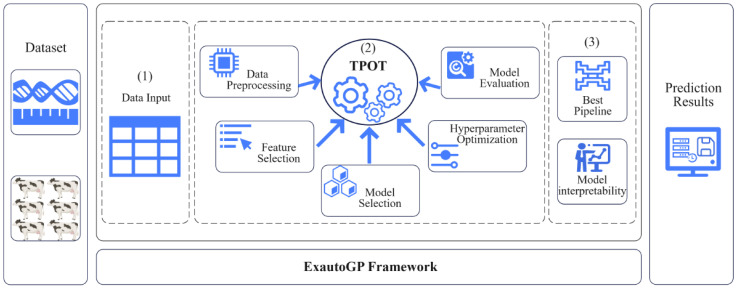
End-to-end workflow of the ExAutoGP framework. It consists of three key components. (1) Data input module. (2) Core TPOT module: Contains components for data preprocessing, feature selection, model selection, hyper-parameter optimization, and model evaluation to automatically and iteratively optimize the ML pipeline. (3) Extraction of the optimal pipeline and interpretation with SHAP.

TPOT [44] is a state-of-the-art AutoML approach proposed by Olson and Moore [22], a framework for automatically designing and optimizing ML pipelines based on genetic programming algorithms, whose operators are mainly derived from the scikit-learn package [45]. The initial phase of TPOT’s work randomly generates a collection of pipelines that contain data preprocessing, feature selection, and model selection steps, and evaluates their performance using cross-validation [22]. Subsequently, TPOT uses the high-performing pipelines as ‘parents’ and generates the next generation of pipelines using a genetic algorithm, which involves crossover (fusion of components from different pipelines) and mutation (random modification of specific parts of the pipeline) to produce ‘offspring’ pipelines. The process involves crossover (fusing different pipeline components) and mutation (randomly modifying specific parts of the pipeline) to produce ‘offspring’ pipelines. Over multiple iterations, TPOT progressively optimizes the performance of the pipeline population through selection, crossover, and mutation. To reduce the risk of overfitting, TPOT draws on an early stopping strategy in ML to monitor the improvement of pipeline performance, and if no significant improvement is seen after a number of generations, the evolutionary process is terminated and the best pipeline is selected [46]. Furthermore, TPOT’s optimization strategy strikes a balance between utilizing the identified optimal pipeline and exploring novel pipeline designs. By automating the end-to-end ML process, TPOT not only significantly saves time and reduces human intervention, but also may discover complex pipeline structures that are inaccessible to manual design, thus increasing the likelihood of generating high-performance pipelines with the ability to generalize over unknown data [47]. Figure 1 shows an overview of the workflow of the ExAutoGP framework implemented based on TPOT.

### 2.3. Evaluation

In this study, we evaluated the performance of all models using a 5-fold cross-validation approach. For this purpose, each dataset was randomly divided into five non-overlapping subsets, four of which were used as the training set and the remaining one as the test set, ensuring that each subset was given one chance to serve as the test set. To further enhance the stability and reliability of the model performance evaluation, this cross-validation process was repeated five times, generating a total of 25 independent results. To quantify the prediction accuracy of the proposed method, we calculated the Pearson correlation coefficients for each trait based on the five repetitions of each 5-fold cross-validation and took the average of the 25 results as the final assessment metrics. The Pearson correlation coefficients were calculated using the following formula:

(9) $$r\left(y_{c},\mathrm{GEBV}\right)=\frac{\mathrm{cov}(y_{c},\mathrm{GEBV})}{\sqrt{\mathrm{var}\left(y_{c}\right)\;\mathrm{var}\left(\mathrm{GEBV}\right)}},$$

where $y_{c}$ is the corrected phenotype.

### 2.4. SHAP: Modelling Interpretation

SHAP is a method for interpreting model predictions based on the concept of Shapley value in game theory [48], which aims to quantify the contribution of features to the prediction of an ML model. The SHAP value is calculated by the following formula:

(10) $$\mathrm{SHAP}\left(i\right)=\sum_{F\in N\setminus \{i\}}\frac{|F|!(|N|-|F|-1)!}{|N|!}\left[f(F\cup \{i\})-f(F)\right],$$

where SHAP(i) denotes the Shapley value of the *i*-th feature, *N* denotes the set of all features, *F* denotes the combination of features with the *i*-th feature removed, f(F) denotes the output of the model on the input feature combination *F*, |N| denotes the number of features in the set *N*, |S| denotes the number of features in the set *S*, and f(F∪{i})−f(F) denotes the cumulative contribution of feature *i*. The significant advantage of SHAP is that it is model-independent, enabling it to be widely applied to a wide range of ML algorithms, including linear models, decision trees, and neural networks, thus intuitively revealing features that play a key role in model prediction. As it is based on the Shapley value in game theory, SHAP systematically calculates the difference in marginal contribution to the prediction result when a feature is included or excluded under all possible feature subsets. This approach not only ensures fairness and consistency in feature contribution allocation but also provides highly interpretable quantitative results.

## 3. Results

### 3.1. Comparison of ExAutoGP with GBLUP and BayesB in Genomic Prediction Accuracy

**Figure 2:**
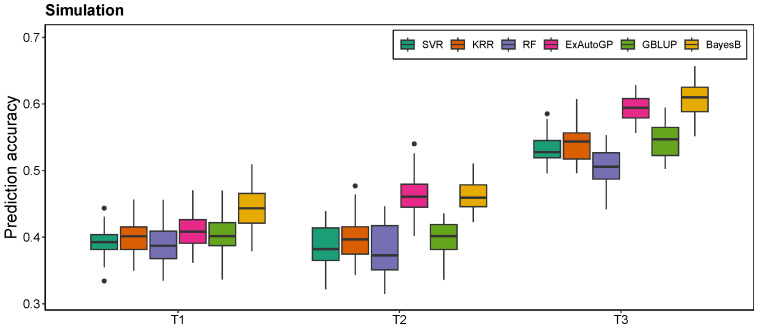
Comparison of prediction accuracies of SVR, KRR, RF, ExAutoGP, GBLUP, and BayesB on simulated datasets. For the box-and-line plot, the line in the middle of the box indicates the median mean correlation, the top of the line extending above the box indicates the maximum value, the bottom of the line extending below indicates the minimum value, and outliers indicate outliers.

**Figure 3:**
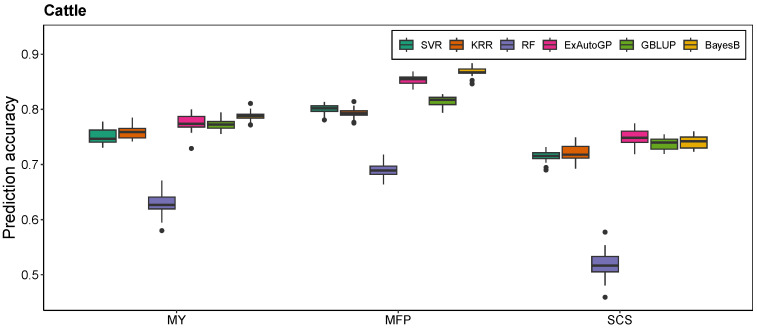
Comparison of the prediction accuracy of SVR, KRR, RF, ExAutoGP, GBLUP, and BayesB on the Holstein cattle dataset. For the box-and-line plot, the line in the middle of the box indicates the median average correlation, the top of the line extending above the box indicates the maximum value, the bottom of the line extending below indicates the minimum value, and outliers indicate outliers.

**Figure 4:**
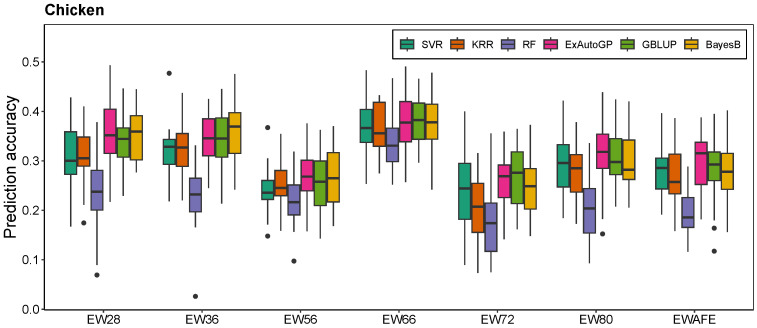
Comparison of prediction accuracies of SVR, KRR, RF, ExAutoGP, GBLUP, and BayesB for the chicken dataset. For the box-and-line plot, the line in the middle of the box indicates the median mean correlation, the top of the line extending above the box indicates the maximum value, the bottom of the line extending below indicates the minimum value, and outliers indicate outliers.

The ExAutoGP method demonstrated a substantial improvement over traditional genomic prediction approaches, namely GBLUP and BayesB, across multiple datasets and phenotypic traits. In the simulated dataset (Figure 2), ExAutoGP achieved prediction accuracies of 0.466 and 0.592 for the T2 and T3 traits, respectively, surpassing the performance of GBLUP (0.397 and 0.545) and BayesB (0.461 and 0.608). Notably, for the T2 trait, ExAutoGP exhibited a 17.4% improvement in predictive accuracy over GBLUP and a 1.1% increase relative to BayesB. However, ExAutoGP is slightly inferior to BayesB in T1 and T3 traits. For the cattle dataset (Figure 3), ExAutoGP achieved a prediction accuracy of 0.854, which was superior to that of GBLUP (0.815), on the MFP trait. In addition, ExAutoGP’s prediction accuracy similarly outperformed GBLUP and BayesB on the SCS trait, while the three had comparable prediction accuracies on the MY trait. On the chicken dataset (Figure 4), for the four traits EWAFE, EW28, EW56, and EW80, ExAutoGP’s prediction accuracy outperformed GBLUP and BayesB to varying degrees, with enhancements ranging from 0.5% to 6.5%. For the EW66 trait, there was no significant difference between the three methods. For the EW36 trait, BayesB performed better and ExAutoGP and GBLUP were comparable. And on the EW72 trait, GBLUP improved by 4% over ExAutoGP, while ExAutoGP improved by 3% over BayesB. Overall, ExAutoGP demonstrated superior and consistent prediction performance on all three datasets. Details of the 5-fold cross-validated Pearson correlation coefficients for five replications are in Supplementary Tables S1–S3.

### 3.2. Comparison of ExAutoGP and Traditional Machine Learning Methods in Genomic Prediction Accuracy

As expected, ExAutoGP significantly outperforms the other three traditional ML methods, namely SVR, KRR, and RF, in all the evaluation datasets. The average prediction accuracy of ExAutoGP over the three datasets is improved by 7.5%, 7.46%, and 28.73% over SVR, KRR, and RF, respectively, with the improvement in each trait ranging from 0.4% to 20.8%, 1.8% to 24.5% and 6.4% to 58.2%. In the simulated dataset (Figure 2), ExAutoGP achieved an average prediction accuracy of 0.489, representing an increase of 11.9%, 9.5%, and 16% compared to SVR (0.437), KRR (0.447), and RF (0.422), respectively. Notably, for the T2 trait, ExAutoGP exhibited substantial improvements over SVR (20.8%), KRR (17%), and RF (23.7%). On the cattle dataset (Figure 3), ExAutoGP showed different degrees of improvement over SVR, KRR, and RF on all three traits, with a 44.6% improvement in ExAutoGP’s prediction accuracy over RF on the SCS trait. On the chicken dataset (Figure 4), for the EW28 trait, ExAutoGP’s prediction accuracy improved by 18.4%, 15.3%, and 53.6% over SVR, KRR, and RF, respectively. Similarly, ExAutoGP improved by 7.6%, 24.5%, and 43.6% over SVR, KRR, and RF on the EW72 trait. For the EW80 trait, ExAutoGP improved the prediction accuracy over RF by up to 58.2%. Detailed information on the 5-fold cross-validated Pearson correlation coefficients for five replications is in Supplementary Tables S1–S3.

### 3.3. Comparison of Computation Time

**Figure 5:**
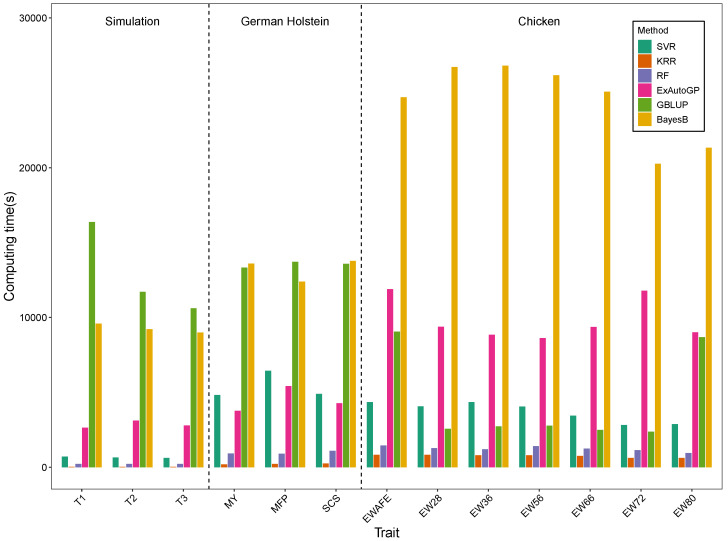
Comparison of computational performance (in seconds) of SVR, KRR, RF, ExAutoGP, GBLUP, and BayesB on three datasets.

We compared the computational time required by the six genomic prediction methods to generate predictions on the three datasets, as shown in Figure 5. The run times are in seconds, and all methods were run on computers equipped with high-performance servers (CentOS Linux 8 operating system, Intel(R) Xeon(R) Gold 5318H CPU @ 2.50GHz processor, 144 CPU cores, and 1TB total memory). To improve efficiency, parallel processing is used for the 5-fold cross-validation of each method. The computational process of TPOT is more time-consuming, as it requires searching for the best machine learning pipeline through continuous iterations. Nevertheless, ExAutoGP runs two to six times faster than BayesB on all three datasets. On both the simulated and chicken datasets, ExAutoGP required more computational time compared to SVR, KRR, and RF. Nonetheless, its fully automated process may mitigate the additional time cost associated with manual hyperparameter tuning. BayesB had the longest average runtime, primarily due to the iterative nature of the Markov Chain Monte Carlo algorithm, which requires extensive iterations to achieve convergence. This issue was particularly pronounced in the chicken dataset, where the substantially larger number of SNPs further exacerbated BayesB’s computational burden. A detailed breakdown of the computational times for each method is provided in Supplementary Table S4.

### 3.4. Interpreting ExAutoGP Model Using the SHAP Method

**Figure 6:**
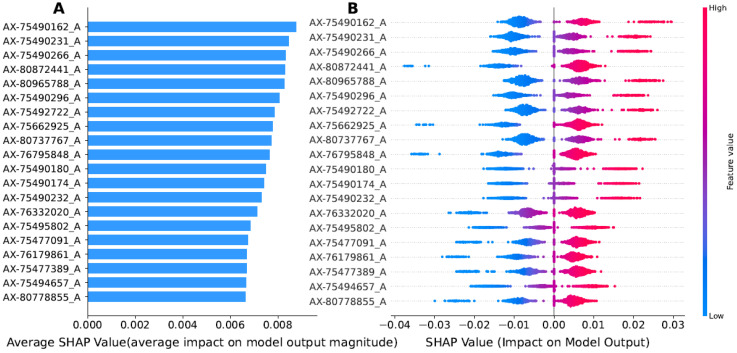
Analysis of EWAFE traits in the chicken dataset using SHAP to assess the importance of features. (**A**) Feature importance ranking: The top 20 traits are sorted in descending order by their mean SHAP values. (**B**) SHAP summary plot showing the distribution of SHAP values for each feature.

We systematically assessed the importance of each trait variable in the ExAutoGP model and its contribution to model prediction using the SHAP method on real traits (see Figure 6 and Figures S1–S9). To illustrate this approach, we present the example of the EWAFE trait from the chicken dataset. On the basis of the model performance evaluation, we further calculated SHAP values to quantify the impact of individual traits on the prediction results. The trait importance bar chart (Figure 6A) illustrates the ranking of trait importance determined based on the mean absolute value of SHAP values, listing the top 20 most important key variables in descending order. These values reflect the average impact of the individual features on the magnitude of the model output. The results show that the AX-75490162_A locus has the most significant contribution among the 20 key loci, followed by AX-75490231_A and AX-75490266_A loci. It is important to note that the feature importance visualization in Figure 6A only includes variables retained after the feature selection process, and all features are displayed with their original nomenclature.

To further parse the decision-making mechanism of the model, we enhanced the interpretability of the results using the SHAP summary graph (Figure 6B). This method arranges individual features around a center line based on their importance. Each point represents a data sample that is labeled at a specific coordinate position based on the SHAP value of its corresponding feature. Features located to the left of the center line have a negative SHAP value, indicating that they have a negative effect on the model predictions, while features to the right have a positive SHAP value, indicating that they drive the model predictions toward a positive outcome. In addition, the color coding of the sample points reflects the magnitude of the feature values, with red indicating higher values and blue indicating lower values. Figure 6B illustrates that the SHAP value distribution for the AX-75490162_A locus is relatively concentrated and positively skewed, indicating a positive contribution to the model output. In contrast, the AX-80872441_A and AX-75662925_A loci exhibit negative contributions, reducing the model’s predicted values. Additionally, the SHAP values of the AX-75490180_A, AX-75490174_A, and AX-75490232_A loci display a narrow range, suggesting a relatively stable influence on model predictions.

According to the study by Liu et al. [32], eight of the top twenty key loci identified using the SHAP method overlapped with SNPs that were previously reported to have significant or suggestive associations with the EWAFE trait. Notably, loci AX-75490180_A (rs314056488) and AX-75490174_A (rs316324927) are located in the intronic region of the candidate gene CECR1 within the chromosomal region associated with cat eye syndrome. In addition, locus AX-75490232_A (rs316675251) is positioned upstream of CECR2, a candidate gene associated with both the EWAFE and EW56 traits. Previous studies have demonstrated that CECR2 plays a crucial role in embryonic development and organogenesis. These findings provide critical insights into the genetic architecture underlying the EWAFE trait and offer valuable directions for further investigation.

## 4. Discussion

In this study, we evaluate the performance of the ExAutoGP method in genomic prediction by comparing it with traditional approaches, including GBLUP and BayesB, as well as ML methods such as SVR, KRR, and RF. The predictive accuracy of ExAutoGP is assessed across multiple datasets to establish its robustness and generalizability. In the simulated dataset, ExAutoGP achieved prediction accuracies of 0.466 and 0.592 for the T2 and T3 traits, respectively, surpassing GBLUP by 17.4% (0.397) and 8.6% (0.545). The genomic prediction accuracies obtained using GBLUP (0.404, 0.397, and 0.545) closely aligned with previously reported values (0.404, 0.400, and 0.545) [49], confirming the reliability of our implementation. Similarly, in the bovine dataset, the prediction performance of the GBLUP and BayesB methods in this study for the three traits was consistent with those reported in the literature [28,50] and slightly worse than that reported by Liang et al. [51]. This discrepancy may be attributed to the differences in the cross-validation strategies used in different studies. Notably, for the milk fat percentage (MFP) trait, ExAutoGP demonstrated superior predictive accuracy (0.854) compared to GBLUP (0.815), highlighting its advantage for high-heritability traits. In addition, the effectiveness of ExAutoGP was further verified in the chicken dataset, where ExAutoGP’s prediction accuracies for the EWAFE and EW80 traits were 0.295 and 0.318, respectively, which were 2.8% and 5.5% higher than GBLUP, and 6.2% and 6% higher than BayesB. However, for some traits (e.g., EW72), GBLUP performed slightly better than ExAutoGP, which may be related to the population structure and the genetic structure of the traits [52].

Comparison with traditional ML methods further highlights the advantages of ExAutoGP. Across all evaluation datasets, the average prediction accuracy of ExAutoGP improved by 7.5%, 7.46% and 28.73% over SVR, KRR, and RF, respectively. In particular, on the T2 trait in the simulated dataset, ExautoGP’s prediction accuracy improved by 20.8%, 17%, and 23.7% over SVR, KRR, and RF, respectively. Notably, the performance of SVR and KRR on the three simulated traits in our study was consistent with the findings reported by Liang et al. [49], while our proposed ExAutoGP model significantly outperformed their method. Furthermore, for the three traits in the cattle dataset, ExAutoGP demonstrated superior predictive performance compared to the improved KRR and SVR models developed by Li et al. [50], and achieved comparable results to the KAML approach proposed by Yin et al. [53]. On the EW80 trait in the chicken dataset, ExAutoGP’s improvement over RF was as high as 58.2%. This significant enhancement in predictive accuracy can be attributed to ExAutoGP’s ability to efficiently capture non-linear relationships and complex patterns in genomic data through automated pipeline optimization. In contrast, traditional ML methods are constrained by the need for manual parameter tuning. Among the three conventional ML models, RF exhibited the poorest performance, primarily due to its sensitivity to hyperparameters such as the number and maximum depth of decision trees. The tuning process for RF is computationally intensive and often fails to yield optimal parameters, further compromising its predictive performance [54]. However, the improvement in prediction accuracy is often accompanied by an increase in computational cost. In this study, we found that although ExAutoGP requires more computational resources on the simulated dataset and the chicken dataset compared to SVR, KRR, and RF, its runtime is still 2 to 6 times faster than BayesB. ExAutoGP’s iterative search strategy based on TPOT is time-consuming, but it avoids the additional time cost of manual parameter tuning through parallel processing and automated processes, thus potentially maintaining a high level of efficiency.

In many areas of scientific research, achieving high predictive accuracy while simultaneously enhancing the interpretability of model decision-making processes is essential. In this study, SHAP analysis was employed to quantify the contributions of individual features to model predictions and to improve interpretability through visualization. The results revealed that the AX-75490162_A locus exerted the most significant influence on model output, with its positive SHAP value distribution further confirming its critical role in prediction outcomes. Moreover, among the SNPs previously reported to be significantly associated with the EWAFE trait [32], eight of the top twenty key loci identified by SHAP analysis in this study were consistent with known loci. These co-identified SNPs play essential roles in the genetic regulation of the EWAFE trait, further reinforcing the reliability of the proposed approach. It is worth emphasizing that the integration of AutoML with SHAP analysis not only enhanced the predictive performance of the model but also improved its interpretability, rendering the decision-making process more transparent. SHAP analysis not only quantifies the importance of individual trait-associated variables and their contributions to model predictions but also facilitates the identification of key genetic markers significantly associated with the trait. This approach enhances the understanding of the genetic regulatory mechanisms underlying complex traits and provides new perspectives for future genetic research.

Previous studies have shown that no single predictive model can be universally applied to all traits due to the complexity and heterogeneity of trait genetic architectures [50,55,56]. To address this challenge, this study introduces AutoML into genomic prediction, offering a novel perspective for improving model adaptability. ExAutoGP leverages TPOT to automate the construction of high-performance ML pipelines, which includes data pre-processing, feature engineering, model selection, and hyperparameter tuning. By systematically and iteratively searching for optimized model architectures tailored to specific datasets and task requirements [19], ExAutoGP effectively captures the diverse association patterns between genotypes and phenotypes. Unlike conventional ML approaches, ExAutoGP dynamically adapts to the genetic background of different traits by automatically evaluating multiple ML models and hyperparameter configurations. This eliminates the reliance on a priori assumptions regarding trait genetic architectures and manual parameter tuning. Furthermore, ExAutoGP significantly reduces the technical barriers for breeders in applying ML by simplifying model development, thereby facilitating its wider application in genomic selection.

Although ExAutoGP demonstrates considerable potential in genomic prediction, it still faces several limitations. On the one hand, ExAutoGP significantly reduces manual intervention and improves modeling efficiency through automated hyperparameter optimization, model selection and feature engineering. However, this automated process requires systematic search and evaluation of a large number of model configurations, which leads to higher computational costs. On the other hand, only three datasets were used in this study, which may have some limitations in terms of model generalization capability. Therefore, future studies may consider introducing more efficient search strategies or utilizing distributed computing techniques to reduce the computational burden and enhance the computational efficiency. Furthermore, the advancement of ExAutoGP should be further validated on larger and more diverse datasets [57], especially in traits regulated by non-additive genetic effects (e.g., dominance vs. epistasis), and it is expected that ExAutoGP will further improve the prediction accuracy of GEBV [58,59]. Notably, an important finding in this study is that multiple key loci significantly associated with the target traits were successfully identified by SHAP analysis, which reveals the decision logic of the model from the perspective of biological mechanisms. This finding highlights the value of introducing interpretable methods into genomic prediction, and also provides an important research direction for further exploring the biological interpretability of models in the future.

## 5. Conclusions

In this study, we introduce ExAutoGP, a novel machine learning strategy for genomic prediction, leveraging the AutoML framework TPOT. Our evaluation of ExAutoGP against five established genomic prediction methods across three datasets demonstrates its ability to dynamically adapt to traits with diverse genetic architectures, delivering robust predictions and consistently outperforming several other machine learning models in prediction accuracy. The integration of the SHAP approach enhances model transparency and enables the identification of key loci associated with target traits, shedding light on the underlying genetic mechanisms and offering valuable insights for breeding strategies. To further advance ExAutoGP, future work will focus on employing more sophisticated search strategies and validating the method on larger and more diverse datasets. In addition, the successful application of SHAP analysis highlights the potential of interpretable approaches in genomic prediction, paving the way for deeper exploration of biological interpretability in predictive modeling.

## Figures and Tables

**Table 1:** Summary of the three datasets.

Dataset	Trait	N	SNPs	$h^{2}$	Mean	SD
Simulation	T1	3000	10,000	0.36	0.00	176.52
	T2	3000	10,000	0.35	0.00	9.51
	T3	3000	10,000	0.52	0.00	0.02
German Holstein	MY	5024	42,551	0.95	370.79	641.60
	MFP	5024	42,551	0.94	−0.06	0.28
	SCS	5024	42,551	0.88	102.32	11.73
Chicken	EWAFE	1052	294,705	0.10	0.00	3.27
	EW28	1063	294,705	0.50	57.19	3.47
	EW36	1063	294,705	0.50	59.15	3.28
	EW56	1027	294,705	0.51	65.83	4.25
	EW66	960	294,705	0.51	80.85	4.34
	EW72	847	294,705	0.54	60.97	3.53
	EW80	852	294,705	0.29	62.33	5.07

**Note**: Trait: the specific phenotype or characteristic being measured. N: number of individuals with the phenotype. SNPs: the number of genetic markers used for analysis. h^2^: heritability of the trait. Mean: the average value of the trait. SD: standard deviation, reflecting the degree of dispersion of trait values.

## Data Availability

The dataset(s) supporting the conclusions of this article is (are) available in the public repository. Simulation dataset: http://qtl-mas-2012.kassiopeagroup.com/en/dataset.php; German Holstein cattle: https://www.g3journal.org/content/5/4/615.supplemental; Chicken: https://figshare.com/articles/Genome-wide_Association_Analysis_of_Age-Dependent_Egg_Weights_in_Chickens/5844420.

## Supplementary Materials

The following supporting information can be downloaded at: https://www.mdpi.com/article/10.3390/ani15081172/s1.

## Abbreviations

The following abbreviations are used in this manuscript:

ML	Machine learning
SHAP	SHapley Additive exPlanations
AutoML	Automated machine learning
SNP	Single nucleotide polymorphism
GEBV	Genomic estimated breeding value
QTL	Quantitative trait locus
GBLUP	Genomic best linear unbiased prediction
SVR	Support vector regression
KRR	Kernel ridge regression
RF	Random forest
TPOT	Tree-based pipeline optimization tool
MY	Milk yield
MFP	Milk fat percentage
SCS	Somatic cell score
EW	Egg weight
EWAFE	First egg weight
EW28	Egg weight at 28 weeks of age
EW36	Egg weight at 36 weeks of age
EW56	Egg weight at 56 weeks of age
EW66	Egg weight at 66 weeks of age
EW72	Egg weight at 72 weeks of age
EW80	Egg weight at 80 weeks of age
