# Supplementary material for: ExAutoGP: Enhancing Genomic Prediction Stability and Interpretability with Automated Machine Learning and SHAP
Source: Animals (Basel). 2025 Apr 18;15(8):1172. doi: 10.3390/ani15081172 (PMC12024354; doi:10.3390/ani15081172)
Supplement: Supplementary file 1 [file animals-15-01172-s001.zip › Figure S1-S9.pdf]

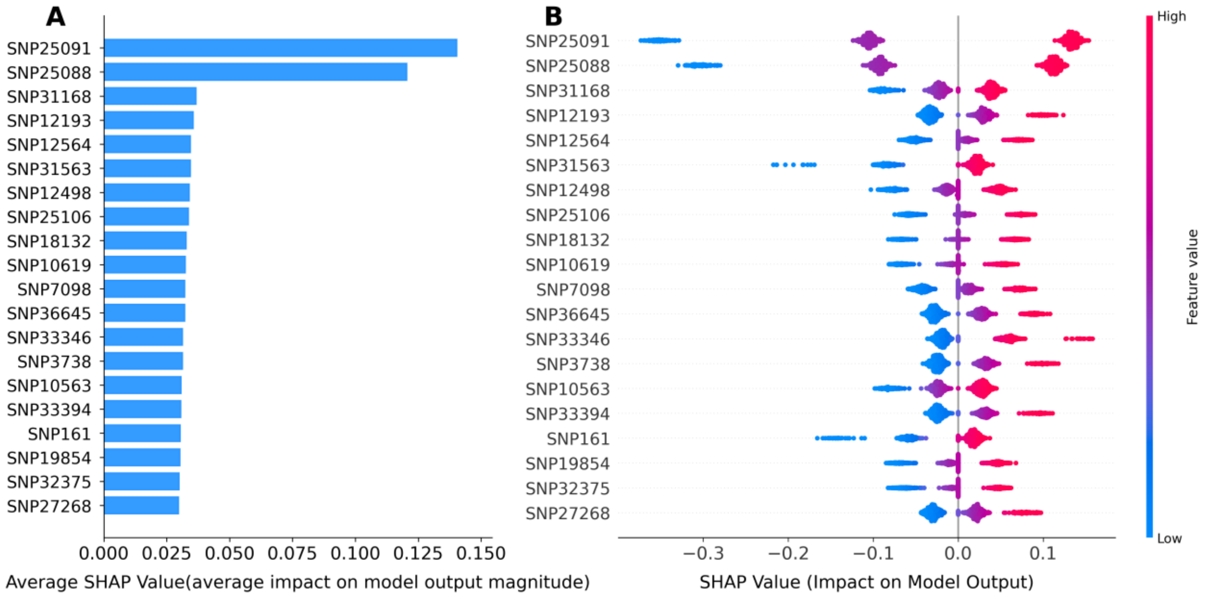

Fig. S1 Analysis of MY traits in the chicken dataset using SHAP to assess the importance of features. (A) feature importance ranking: the top 20 traits are sorted in descending order by their mean SHAP values. (B) SHAP summary plot showing the distribution of SHAP values for each feature.

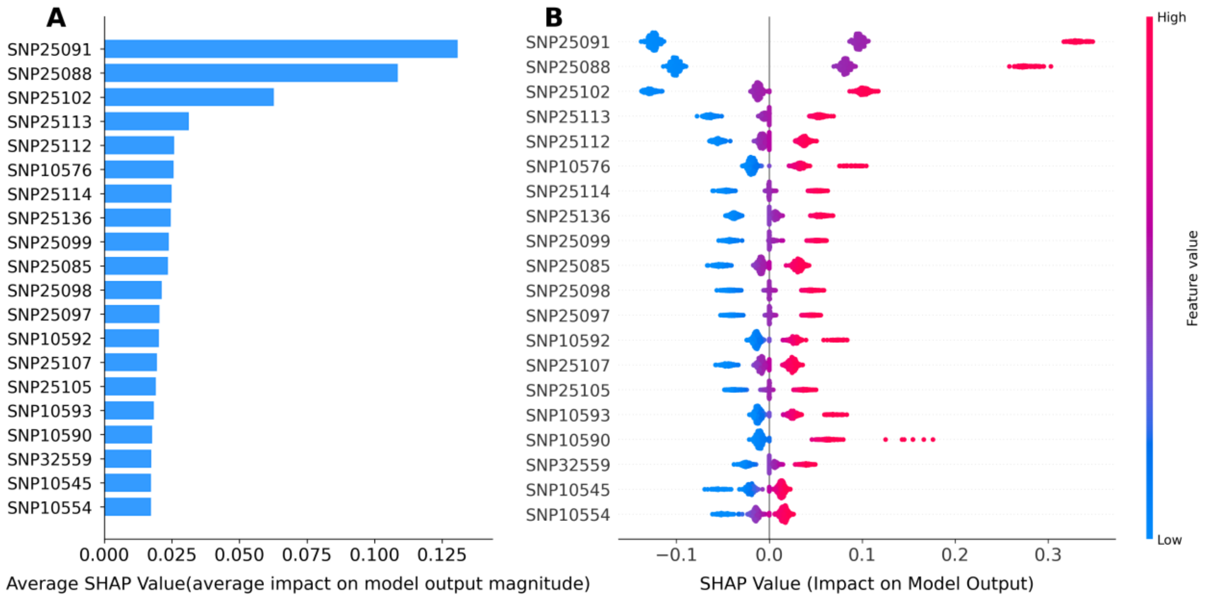

Fig. S2 Analysis of MFP traits in the chicken dataset using SHAP to assess the importance of features. (A) feature importance ranking: the top 20 traits are sorted in descending order by their mean SHAP values. (B) SHAP summary plot showing the distribution of SHAP values for each feature.

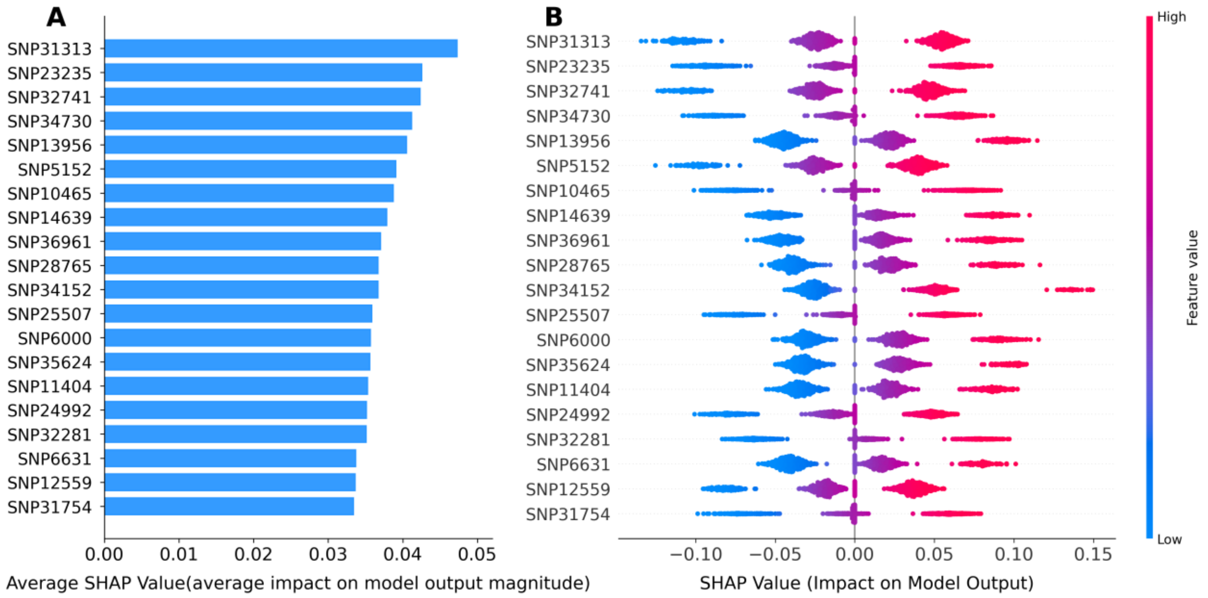

Fig. S3 Analysis of SCS traits in the chicken dataset using SHAP to assess the importance of features. (A) feature importance ranking: the top 20 traits are sorted in descending order by their mean SHAP values. (B) SHAP summary plot showing the distribution of SHAP values for each feature.

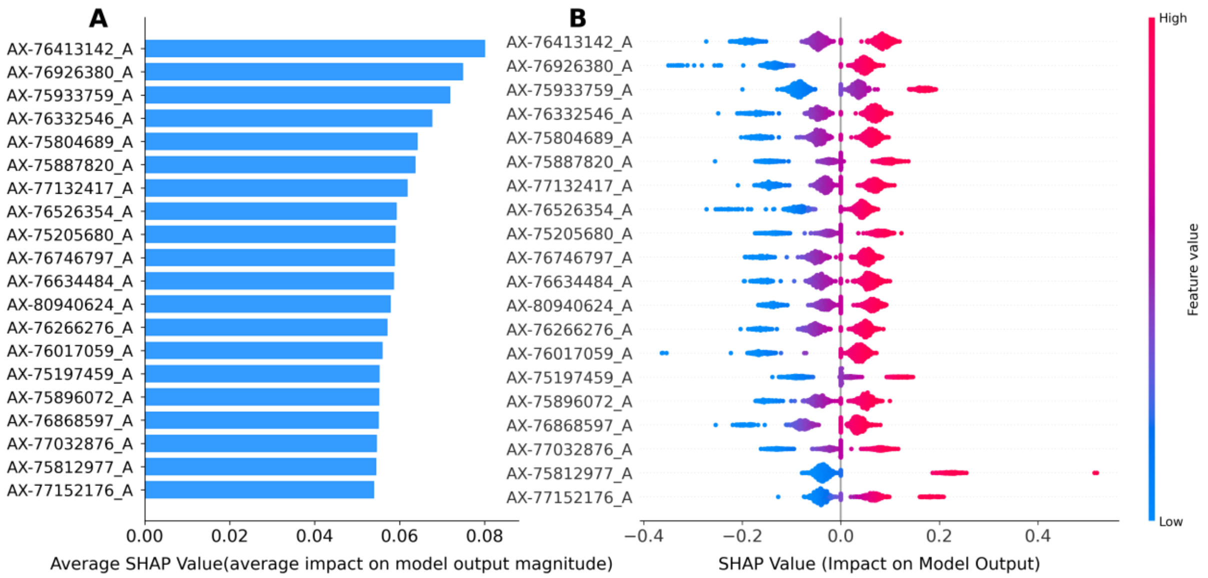

Fig. S4 Analysis of EW28 traits in the chicken dataset using SHAP to assess the importance of features. (A) feature importance ranking: the top 20 traits are sorted in descending order by their mean SHAP values. (B) SHAP summary plot showing the distribution of SHAP values for each feature.

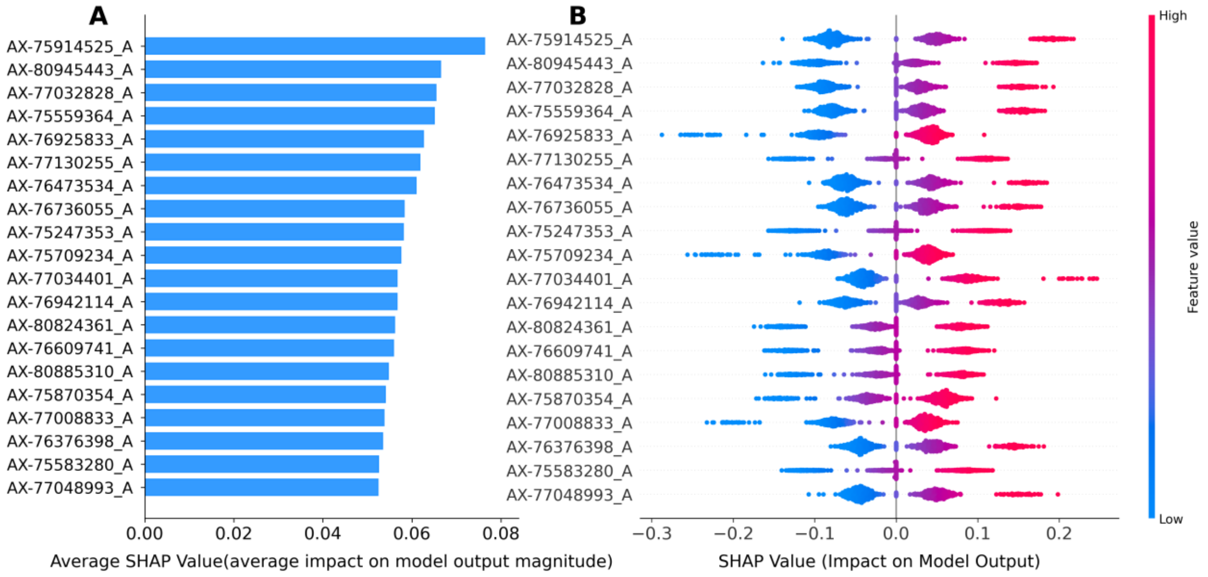

Fig. S5 Analysis of EW36 traits in the chicken dataset using SHAP to assess the importance of features. (A) feature importance ranking: the top 20 traits are sorted in descending order by their mean SHAP values. (B) SHAP summary plot showing the distribution of SHAP values for each feature.

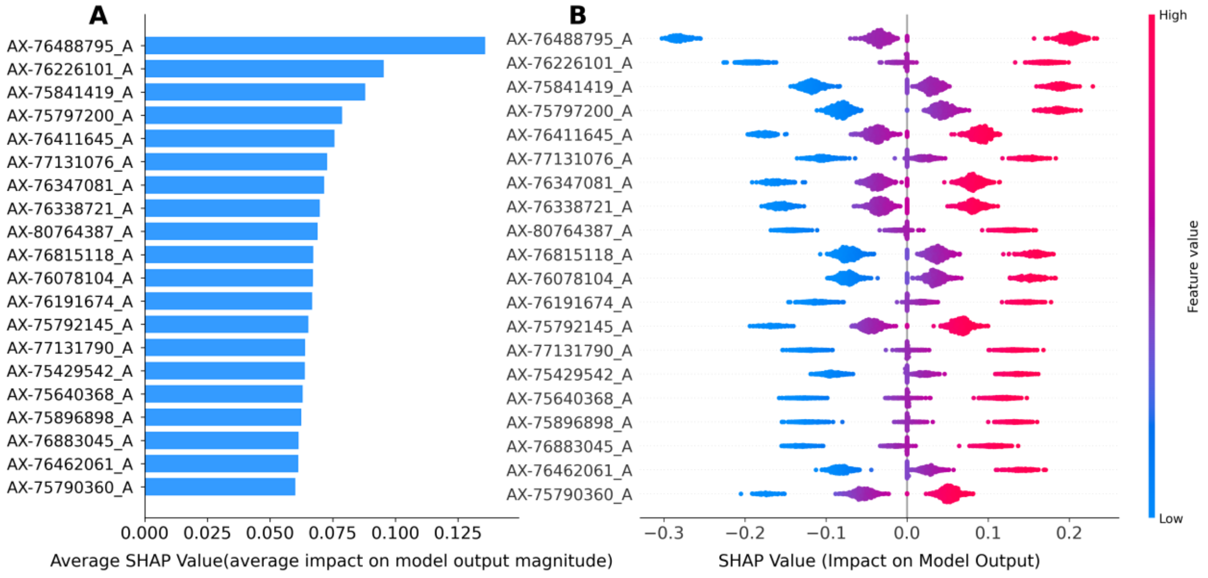

Fig. S6 Analysis of EW56 traits in the chicken dataset using SHAP to assess the importance of features. (A) feature importance ranking: the top 20 traits are sorted in descending order by their mean SHAP values. (B) SHAP summary plot showing the distribution of SHAP values for each feature.

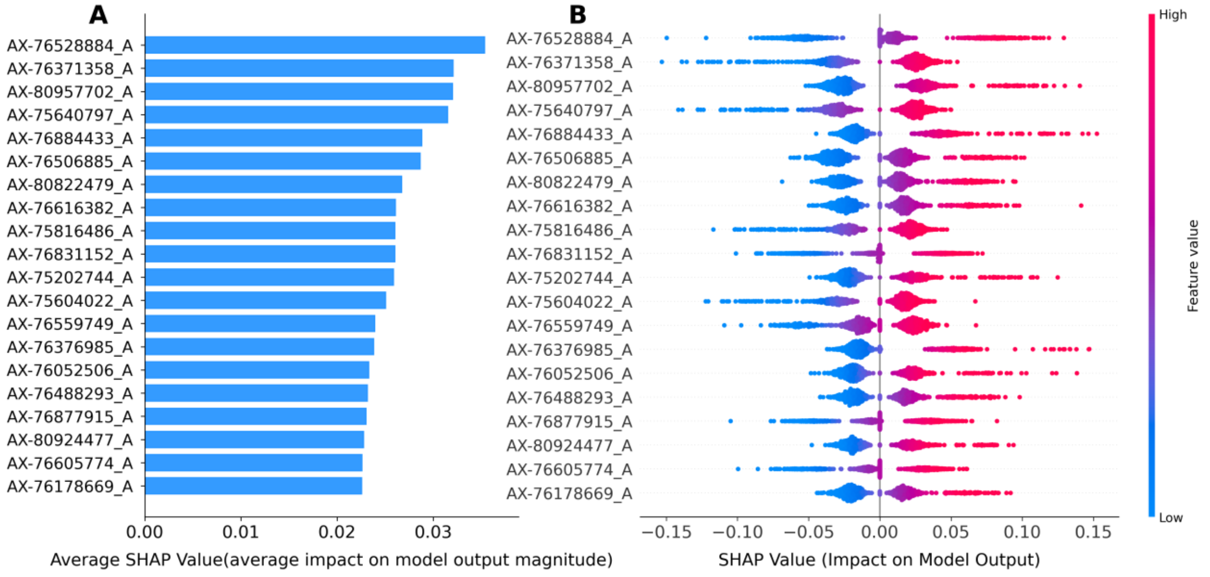

Fig. S7 Analysis of EW66 traits in the chicken dataset using SHAP to assess the importance of features. (A) feature importance ranking: the top 20 traits are sorted in descending order by their mean SHAP values. (B) SHAP summary plot showing the distribution of SHAP values for each feature.

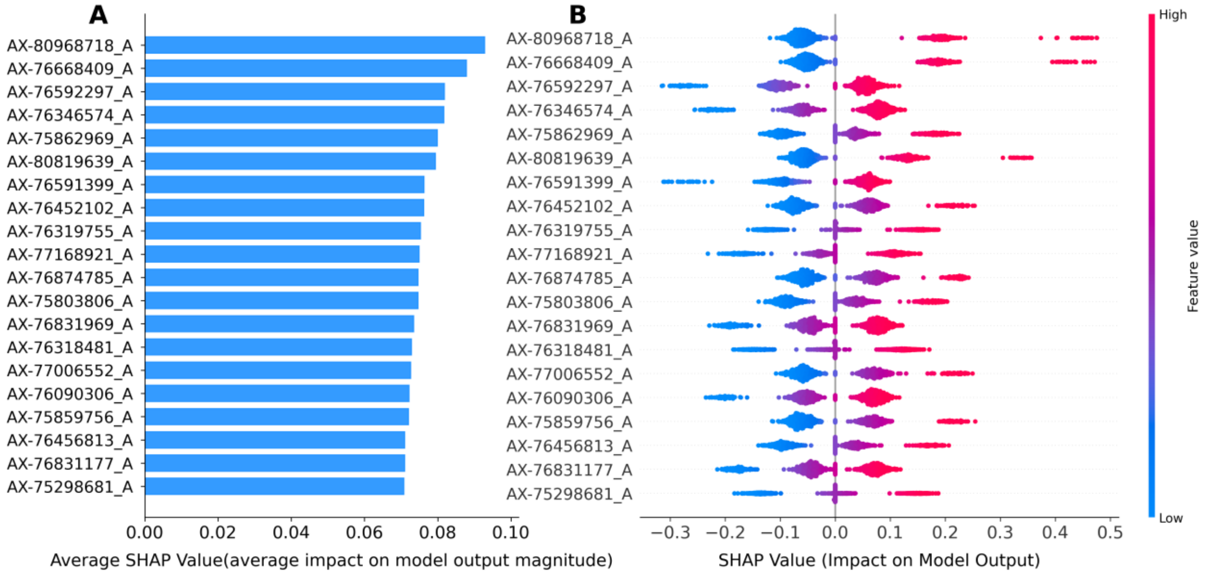

Fig. S8 Analysis of EW72 traits in the chicken dataset using SHAP to assess the importance of features. (A) feature importance ranking: the top 20 traits are sorted in descending order by their mean SHAP values. (B) SHAP summary plot showing the distribution of SHAP values for each feature.

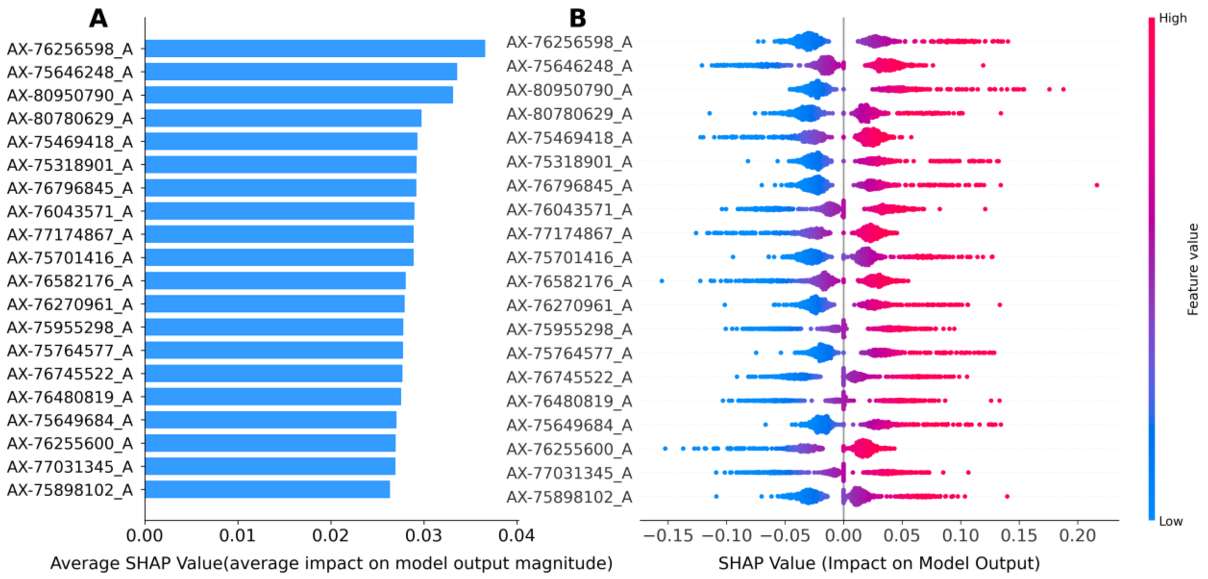

Fig. S9 Analysis of EW80 traits in the chicken dataset using SHAP to assess the importance of features. (A) feature importance ranking: the top 20 traits are sorted in descending order by their mean SHAP values. (B) SHAP summary plot showing the distribution of SHAP values for each feature.
